# Learning autonomy in two or three steps: linking open-ended development, authority, and agency to motivation

**DOI:** 10.3389/fpsyg.2013.00766

**Published:** 2013-10-22

**Authors:** Tjeerd C. Andringa, Kirsten A. van den Bosch, Carla Vlaskamp

**Affiliations:** ^1^Artificial Intelligence and Cognitive Engineering (ALICE), University of GroningenGroningen, Netherlands; ^2^Special Needs Education and Youth Care, University of GroningenGroningen, Netherlands

**Keywords:** motivation, agency, autonomy, open-ended development, co-creation, authority, complexity, lateralization

## Abstract

In this paper we connect open-ended development, authority, agency, and motivation through (1) an analysis of the demands of existing in a complex world and (2) environmental appraisal in terms of affordance content and the complexity to select appropriate behavior. We do this by identifying a coherent core from a wide range of contributing fields. Open-ended development is a structured three-step process in which the agent first learns to master the body and then aims to make the mind into a reliable tool. Preconditioned on success in step two, step three aims to effectively co-create an optimal living environment. We argue that these steps correspond to right-left-right hemispheric dominance, where the left hemisphere specializes in control and the right hemisphere in exploration. Control (e.g., problem solving) requires a closed and stable world that must be maintained by external authorities or, in step three, by the right hemisphere acting as internal authority. The three-step progression therefore corresponds to increasing autonomy and agency. Depending on how we appraise the environment, we formulate four qualitatively different motivational states: submission, control, exploration, and consolidation. Each of these four motivational states has associated reward signals of which the last three—successful control, discovery of novelty, and establishing new relations—form an open-ended development loop that, the more it is executed, helps the agent to become progressively more agentic and more able to co-create a pleasant-to-live-in world. We conclude that for autonomy to arise, the agent must exist in a (broad) transition region between order and disorder in which both danger and opportunity (and with that open-ended development and motivation) are defined. We conclude that a research agenda for artificial cognitive system research should include open-ended development through intrinsic motivations and ascribing more prominence to right hemispheric strengths.

## Introduction

In this theoretical paper we aim to unify a number of complementary and highly consistent results from a wide range of scientific domains that *all* pertain to “learning to cope autonomously with the challenges of an open environment.” We will frame these results in terms of agency and autonomy development. In the final section we will formulate what we call the “open-ended development loop” (Figure 5) as a main and productive synthesis for artificial cognitive system research and behavioral sciences in general.

In our efforts we benefitted from results and insights from life-span research, personality development, emotion theory, psychoanalysis, motivation research, brain lateralization, political psychology, soundscape research, complexity theory, and even early Chinese philosophy. In addition, although in this paper less prominent, we benefited from moral psychology, epistemological development, and education research. While this may seem an unnecessary wide range of scientific domains to address the call-topic of “open-ended development driven by intrinsic motivations” we argue that both the concepts of “open-ended development” and “motivation” are not just cognitive functions, but cognitive foundations: without motivation there would be no activity and no agency.

As cognitive foundations, “motivation” and “open-ended development” shape and constrain many facets of cognition. As such, insights from all specialisms of the cognitive sciences in the broadest sense, and in particular those domains directly related to open environments, may contribute with novel perspectives on foundational principles. We will outline that open-ended development and motivation are intimately related with concepts such as agency, mood, behavior, and action selection, brain lateralization, appraisal, safety, and complexity. In addition we will introduce the terms “authority” (defined as the capacity to create, maintain, and influence living environments), and “co-creation” (defined as the ability to work with the inherent dynamics of the world instead of suppressing and controlling it) as fundamental concepts for understanding agency and cognition.

Since we derive from many domains of science we focus more on the relations between relevant concepts and the progression of argument than on experimental or implementation details. In many cases we will slightly generalize domain specific terms, concepts, and results to make them more consistent with each other. Our inductive approach to science was only possible because of the many deep and precisely formulated insights by researchers from very different traditions, which strengthens our belief that the true value of scientific insights can only be estimated outside of the domain where it was developed. We present many of these insights as direct quotes so that the quality of the formulation can also be appreciated in a quite different context than the original publication.

Our paper has, apart from the introduction, 4 main sections. In section Open-Ended Development we address a wide range of results and insights consistent with the title of our paper, suggesting that open-ended development occurs in two or three steps, with the third step being pre-conditioned on the success of the second. In step one the agent's focus is on making the body into a reliable instrument. The second step involves making the mind into a reliable and effective tool. Only success in this step allows a third phase in which the agent learns to shape—co-create—the conditions for its continued existence and in doing so it becomes independent of external authority and truly autonomous. We visualize this two or three step approach as a spiral development in which matching development phases stemming from diverse fields of research have been indicated. This spiral epitomizes open-ended development.

In the next section we address two attitudes toward a complex world. One associated with exploration and the other with control. We couple these attitudes to two modes of being and understanding of the world that comply very well with the different strengths of the left (control) and right (exploration) hemisphere. Here we conclude that step one and three rely on right hemispheric dominance and step two on left hemispheric dominance. We couple this conclusion to a need of external authority associated with a dominant left hemisphere.

In section Motivations we address motivations by first focusing on some of our own results that couple four qualitatively different appraisals of the (sonic) environment to motivational states in the context of core affect. We argue that each of the four quadrants of core affect constrains mind-states in a distinct way and that motivation can be treated as attitudes toward particularly appraised worlds. We end this section with a table describing these four quadrants in terms of motivation and other properties derived from different scientific domains.

In section Open-Ended Development Driven by Intrinsic Motivation we address the call topic “open-end development driven by intrinsic motivations” by outlining the conditions required for open-ended development, which, we argue, rely essentially on the agent learning to shape its own environment. We argue that the results of motivation research, interpreted in the context of the four quadrants, describe what we call the “open-ended development loop.” We note a number of observations and constraints to be satisfied for open-end development to occur that might be used to formulate a research agenda for artificial cognitive systems research. We end with the observation that particular Western—left hemispheric—biases have limited our understanding of cognitive systems and we suggest a way to address these limitations.

## Open-ended development

**Figure 1:**
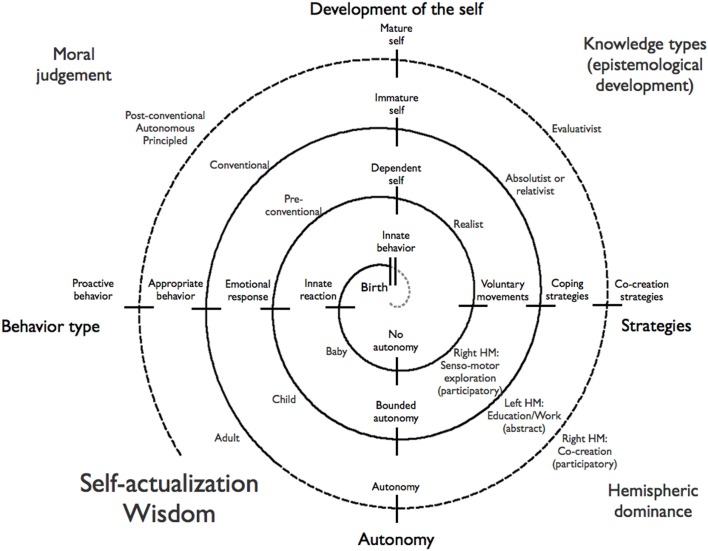
**Open-ended development**. This spiral development depicts phases in open-ended development and terms typically associated with different development phases. The inner rotation can be described as learning to master the body, the second rotation as making the mind a reliable tool and the third learning to effectively co-create an optimal living environment. We propose that this corresponds to a progression from right to left to right hemispheric dominance and associated strategies. [Inspired by the depiction in Arnold, (1910), p. 23].

Open-ended development is not undirected, quite the contrary. The research outlined below shows that open-ended development refers to the capacity to ever-extend and fine-tune one's capacity to deal with life's challenges and to co-create one's environment. Put differently: open-ended development is a development that allows agents to gradually master more and more of the complexity of the world and to become more and more self-deciding, agentic, and autonomous. Figure 1 visualizes open-ended development and it summarizes many of the results that we address in this section in terms of reported stadia of open-ended development. While this section addresses the many properties of open-ended development, its main drivers—the demands of an open world, (intrinsic) motivation, and the open-ended development loop—will be addressed in later sections.

The spiral development outwards makes about three turns that reflect, very roughly, three developmental phases. The first phase is physical growth and learning to control the body. In the second phase one aims to makes the mind into a reliable instrument. The third phase, preconditioned on the success of phase 2, concerns learning to co-construct a world in which the inherent dynamics of the world are stabilized and made reliable and broadly beneficial. This leads to ever more extended (both in place and in time) environments in which one can self-maintain the condition for adequate functioning, leading to increasing diversity and individual authority. This characterizes the outer (pre-conditioned) loop of the spiral development in Figure 1.

The figure has a number of functional components. The spiral is divided into a number of sectors that reflect aspects of open-ended development without being necessarily in the strict circular progression the spiral form suggests. The end-state of the spiral is referred to as self-actualization or wisdom. The solid-line part of the spiral reflects development up the level of the authoritarian personality, while the dashed part reflects an—in Western cultures non-standard—additional development toward the libertarian personality type. The main axes reflect behavior types and strategies horizontally and self-development and action readiness vertically. The diagonal axes reflect distinct development stages from diverse scientific fields: moral development, education research and epistemological development, and brain lateralization research. In the next subsections we'll provide supportive evidence for each axis and gradually develop the key terminology of this paper.

### End-state: self-actualization and wisdom

Open-ended development in humans is a highly structured process that has been well studied in a variety of different domains that each shed more light on the phases in the development process. The development process begins obviously at conception and develops after birth in a number of stages toward what Maslow (1943, 1962) calls self-actualization. According to Maslow, self-actualization accounts for the highest possible forms of psychological health and self-development. As such it is a candidate for fully developed open-ended learning. Among the main characteristic properties of a self-actualized individual are (1) realistic perceptions of themselves, others, and the world around them, (2) a strong motivation, through a sense of personal responsibility and ethics, to help others and to find solutions to problems in the external world, and (3) a well-developed personal autonomy, which is for example visible as an utter disregard of conformity if the situation demands this and an appreciation for private time to self-develop one's potential further.

Compared to not (yet) self-actualized individuals they

1. Have learned the skills to prevent or overcome one's own psychological problems that allow them to be rarely motivated by unfulfilled needs,
2. Have developed a deep and pervasive understanding of reality that they keep extending through life and that is apparent from a well-developed creative capacity to produce intended results with minimal adverse side-effects, and
3. Feel a moral obligation to contribute to an improved world.

These properties reflect deep realities concerning the nature of agentic life. Interestingly the term self-actualization arose from Maslow's work on motivation (Maslow, 1943), but he refined and defined the term self-actualization later on the basis of case-studies of individuals of whom he thought that they represented examples of self-actualization (Maslow, 1962). This intuition-driven (dangerously circular) process is vindicated by results later in this paper that dovetail with Maslow's conclusions while being based on entirely different evidence.

Another way to approach open-ended development comes from gerontology and especially the role of lifelong learning and continued education for older people which allows them to stay involved in a rapidly changing world (Ardelt, 2000). This led to a distinction between intellectual knowledge and wisdom-related knowledge, of which the wisdom related knowledge develops on a basis of intellectual knowledge. Wisdom-related knowledge inductively reduces the quantity and complexity of intellectual knowledge in favor of what is deeper and more essential. Wisdom researcher Sternberg defines wisdom as follows:

“*the application of tacit knowledge towards the application of a common good through a balance among intra-, inter-, and extra-personal interests to achieve a balance among adaptation to existing environments, shaping of existing environments, and a selection of new environments, over the long term as well as the short term.*” (Sternberg, 1998)

One might summarize wisdom as “the ability to produce broadly beneficial intended results while taking the full consequences of behavior into account.” Again we find a combination of skill (tacit knowledge), and (implicitly) a pervasive (long term) understanding of reality, in combination with an urge to improve and shape the living environment. We consider this developing urge to improve and shape living environments an essential aspect of open-ended development and propose an explanation for that below in the section on a complex world.

### Authoritarians and libertarians

The solid part of the spiral is the development up to the level of the authoritarian personality as defined by Stenner (2005, 2009). Authoritarians “*are not endeavoring to avoid complex thinking so much as a complex world* (Stenner, 2009, p. 193).” It is the authoritarian's underdeveloped cognitive capacity that “*reduces one's ability to deal with complexity.*” This personality-type seeks, appreciates, and even demands external authorities to maintain the living conditions in which they can function adequately: normalcy. For authoritarians “authorities” are the processes or agents that they perceive as responsible for maintaining normalcy (and with that their sense of adequacy). Authoritarians display “bounded autonomy” because they exhibit autonomy only in a suitably controlled environment. Authoritarians actively help their authorities in a particular and highly characteristic way: by reducing the perceived complexity of the environment; in particular through intolerance of diversity and by supporting some perceived central authority (an agent or process) with the same surmised aim.

The dashed part of the spiral progresses beyond this level to the libertarian personality (Stenner, 2005). Libertarians have gradually developed the autonomy and skills to co-create living conditions in which they and others feel and act adequately without the need for external authority to maintain and create these conditions. Libertarians have internalized the role of authority and prefer therefore individual authority to centralized authority. As such libertarians become local centers of development and growth in their (social) environment and consequently centers of diversity. Compared to authoritarians who can function adequately in standard situations and tend to exhibit norm-complying and norm-returning behavior, libertarians (have learned to) understand the world to a degree that they can cope effectively with deviations from normalcy and they use the benefits this provides to enhance their lives.

Stenner used a very simple “child-rearing values test” (Stenner, 2005) to determine whether individuals were authoritarian or libertarian (she only used the extremes in her analysis). Participants that clearly preferred children to be raised as obedient conformist were deemed authoritarian and those that preferred children to be raised as independent self-deciders were deemed libertarian. Apparently this simple six two-option test was enough to separate people into a group that aims to avoid (a more) complex world and a group that can comfortably deal with some more complexity. Stenner specifically identifies the reaction to “normative threads,” perceptions of leadership failure and diversity in public opinion, as key difference between authoritarians and libertarians.

Authoritarian behavior depends on whether or not the situation might develop beyond coping capacity. This entails that *“individuals with a certain level of authoritarianism may manifest entirely different attitudes and behaviors from one occasion to the next, depending upon the presence or absence of normative threat* (Stenner, 2009, p. 189).” And “*normative threat only invites this kind of fear, cognitive unraveling and out-bursts of intolerance among authoritarians, whereas in fact these very same conditions (i.e., the public dissension and criticism of leaders that are the hallmarks of a healthy democracy) induce only greater tranquility, sharper cognition, and more vigilant defense of tolerance among libertarians* (Stenner, 2009, p. 193).” We will use this observation in the next section to differentiate between Two Attitudes Toward a Complex World.

### Main axes

The axis from the center leftward in Figure 1 reflects increasingly more advanced responses to environmental challenges developing from innate (e.g., sucking), via emotional (e.g., happy or frustrated), to appropriate (e.g., culturally sanctioned) and even proactive responses (e.g., preventing future problems or creating a better society). Protruding downward is an axis denoting autonomy development. This axis develops from no autonomy at all, via the bounded autonomy of authoritarians, to the autonomy of libertarians. Extending rightward is an axis reflecting strategies developing from voluntary movements and direct perception-action relations, via coping strategies for the here and now, to advanced co-creating strategies that define and shape the environment (i.e., the agent as authority).

The axis extending from the center upwards reflects a development from a dependent self, to an immature and mature self. This development of the self has two separate but related facets: social and personal maturity. “*Social maturity is defined by measures of adaptation such as life satisfaction, environmental mastery, or positive social relations. Personal maturity, however, is indexed by openness to experience and indicators of personal wisdom such as personal growth and ego development* (Staudinger and Glück, 2011, p. 213).” Development of the self moves people increasingly away from egocentric, dependent, and self-centered modes of being (in Figure 1 referred to as “immature self”), toward the capacity to take perspectives on the self and others, and to experience positive, helpful, responsible, and mutual interaction with others referred to as “mature self” (Richardson and Pasupathi, 2005, p. 145).

### Diagonal axes

The lower left diagonal in Figure 1 simply reflects the development from a baby, which is preoccupied with discovering its body and its immediate environment, to childhood in which it is preoccupied with the exploration of the neighborhood and the acquisition of habits, skills, and knowledge, and to adulthood in which one's potential can be developed and utilized in full.

The upper left diagonal shows the three main stages of moral development as described by Kohlberg (1971). Kohlberg calls the first main stage “pre-conventional” in which *the child only understands the consequences of its behavior in terms of direct effects on self in terms of (un)pleasantness and in which it knows that obedience is a way to avoid punishment*. At this stage *right action concerns mainly the satisfaction of one's needs*. In the second, “conventional,” phase *the individual's attitude is not only one of conformity to personal expectations and social order, but of loyalty to it. It actively maintains, supports, and justifies the order and identifies with the persons or group involved in it.* This phase corresponds closely to the description of authoritarianism. The third stage is called the “post-conventional,” “autonomous,” or “principled” level. Individuals at this stage *make a clear effort to define moral values and principles that have validity and application apart from the authority of the groups of persons holding them and apart from the individual's own identification with the group* (Kohlberg, 1975). This stage corresponds closely to the description of libertarians. A 20-year longitudinal study in Chicago found moral judgment development to be positively correlated with age, socio-economic status, IQ, and education. In addition development in childhood predicted development in adulthood. At age 36 only about 10% had reached a moral development at post-conventional level (Colby et al., 1983); this suggests indeed that it is more an option than a default in modern Western cultures.

The upper right diagonal reflects words from the field of epistemological development [see van Rossum and Hamer (2010) for an overview] and in particular from Kuhn et al. (2000) who separates four levels of beliefs about the world. In the first “realist” level, assertions exist only in direct reference to a state of the world. In the second “absolutist” level assertions are authority derived true or false representations of the world. In the third level assertions are opinions that can be freely chosen, are accountable to their owners, and that, apart from authority support, cannot be ranked in terms of quality. In the fourth level assertions are judgments that can be evaluated and compared according to criteria of argument and evidence. This fourth level has passed what van Rossum and Hamer (2010), p. 26 call the watershed between reasoning in terms of ready-made things (facts, procedures) existing “out there” to independently constructing meaning. Since this is, again, a transition between dependence and independence of authority we associate (but not equate) the “watershed” with the transition from authoritarianism to libertarianism.

The last diagonal, in the lower right, describes typical activities associated with different life-phases. A baby is typically involved in all forms of sensory-motor explorations in which it gradually learns to separate the whole of perceptual and motor experiences into meaningful units. This parts-from-whole approach of participatory discovery is typically associated with the right brain hemisphere (McGilchrist, 2010). The second phase is typically culturally, technically, and representationally driven. In this phase the main sources of knowledge are represented and conveyed via languages (of diverse forms) and technologically and culturally constructed objects and environments. This is a phase in which—in our Western cultures—the left hemisphere is dominant. It is also a world in which knowledge and skills are constructed from parts-to-whole. Knowledge and skills are typically not self-discovered but directly derived from others (authorities). In the post-watershed phase the participatory co-creation that characterizes self-actualized individuals takes again the effect of behavior in an ever-extending context into account. This suggests a return to right hemispheric dominance. The processes that drive these developments, and the rational to assign them to dominant hemispheres will be addressed in the next section.

The next section addresses two essentially distinct modes of being that are believed to underlie both hemispheric differences as well as the key properties of intrinsic and extrinsic motivations that, we claim, differ in the way they approach a complex situation.

## Two attitudes toward a complex world

Complexity research has shown (Kauffman, 1995; Capra, 1997) that all life and therefore all human activity seems to occur in the transition region between order and disorder or structure and chaos (Mora and Bialek, 2011). Too much structure precludes diversity and development. Too much disorder precludes stability and predictability. Put differently: moderately increasing disorder allows for more diversity and development but allows less control. In moderation, disorder may lead to novelty, in excess it leads to chaos. In contrast, increasing order fosters uniformity, predictability, and control, but in excess it leads to stagnation and lifelessness. Note that the moment a novel structure has been discovered in a previously disordered or chaotic state, some order (and meaning) is imposed on it and the complex system becomes a little more tractable and accessible to agent influence. With this discovery the “edge of chaos” has been pushed toward higher complexity. We propose that this process pushes development along the spiral in accordance with Vygotsky (1978) zone of proximal development.

### Two modes of cognition

We can call the form of cognition that allows us to discover novel structure “cognition for disorder,” “cognition for possibilities,” or “explorative cognition.” Whatever it is called, its essential nature is participatory: structures in (apparent) chaos are only discovered through some form of participation in the system. During exploration and play, the properties of these structures are revealed and the structures of interest become gradually more familiar and predictable. This allows their properties to be generalized, abstracted, and integrated with existing knowledge and in doing so made useful for in the widest possible range of environments and (individual) challenges.

In situations where errors are costly (or even deadly) we need a complementary form of cognition: a form that more aptly is called “cognition for order,” “cognition for certainty,” or “control cognition.” Both are essential forms of cognition and together they allow for a gradual proven and reliable extension of the limits of agent capability toward ever more complex situations and ever-larger temporal and spatial scopes. This continual progression of exploration, consolidation, and testing is another formulation of open-ended development.

Recall that the reaction to an increasing complex world is the key difference between authoritarians and libertarians (Stenner, 2009). This suggests that the complexity of our (living) world is a deciding factor in determining whether someone is (or behaves as) authoritarian or libertarian. Authoritarians tend to abhor a complex world and feel an urge to reduce its complexity, while libertarians can deal comfortably with some additional complexity. The authoritarian reaction to increased complexity is with fear and intolerance of diversity (reducing complexity), while the libertarian reacts with increased interest and sharper cognition (mastering complexity). This suggests that explorative cognition and control cognition, in particular with authoritarians, are activated depending on whether the environment is appraised as safe or unsafe.

**Figure 2:**
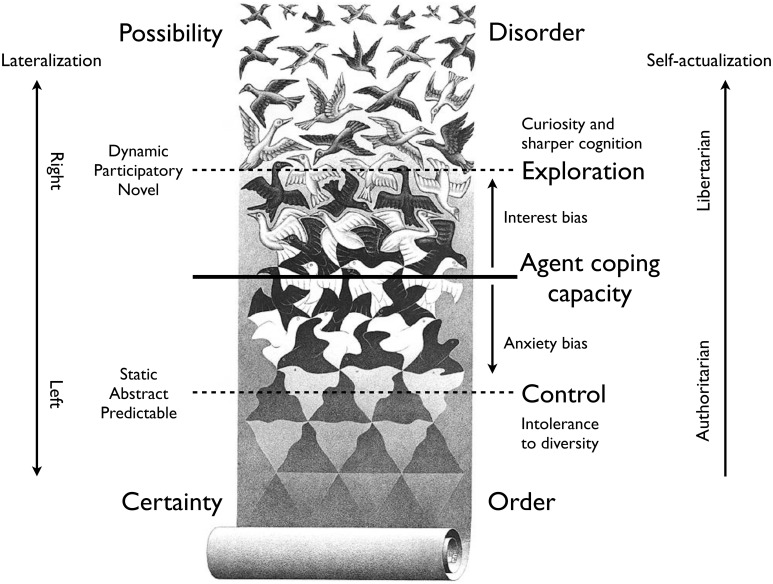
**Dealing with complexity**. The anxiety-free response to increased complexity leads to curious exploration and sharper cognition, while the anxiety-laden response activates intolerance of diversity. This graphical depiction can be interpreted as agent development at some part of the spiral in Figure 1 that gradually moves outwards toward self-actualization. (M.C. Escher's “Liberation” © 2013 The M.C. Escher Company—the Netherlands. All rights reserved. Used by permission. www.mcescher.com).

The depiction in Figure 2 visualizes these two cognitive responses. The backdrop is Escher's, 1955 tessellation “Liberation” that reflects a progression from lifeless, predictive structure toward living free dynamics and endless possibilities. Here we assume that an agent's coping capacity allows it to deal with some intermediate level of complexity half way this progression. Depending on whether the overall situation is perceived as safe or unsafe, an agent might be motivated to explore dynamic diversity and novelty—the interest bias—or be motivated to reduce the complexity of the environment by helping to reduce the complexity through curtailing diversity and dynamics—the fear bias. The higher the life-fraction spent with an interest bias, the more one explored and the more one learned to master complexity.

It is therefore not surprising that the personality trait “openness to experience” correlates positively with libertarianism (Stenner, 2005). According to McCrae and Sutin (2009) “*highly open people are thus seen as imaginative, sensitive to art and beauty, emotionally differentiated, behaviorally flexible, intellectually curious, and liberal in values. Closed people are down-to-earth, uninterested in art, shallow in affect, set in their ways, lacking curiosity, and traditional in values.*” This contrast reads as a preference for an interesting vs. an ordered world. In addition “*open people admire openness, closed people despise it* (McCrae and Sutin, 2009).” Associated with a closed attitude is “the need for closure” (Kruglanski and Webster, 1996; Malhotra et al., 2008), the desire for definite and final answers. People prone to seizing on the first idea offered and then freezing on this solution are in general uninterested in exploring alternative possibilities, keeping their views simple and uncluttered.

### Two hemispheres

The existence and detailed properties of these two forms of cognition have recently been described in the seminal work on the divided brain by McGilchrist[2010; see Rowson and McGilchrist (2013) for a highly accessible introduction]. McGilchrist argues that the two cortical hemispheres understand the world in quite different ways. In particular it suggests to us that the left hemisphere specializes in cognition for order, while the right hemisphere specializes in cognition for disorder. Table 1 provides a representative fraction (McGilchrist, 2010 chapter 1) of the wealth of reported differences in *how* the individual hemispheres approach and understand the world.

**Table 1:**
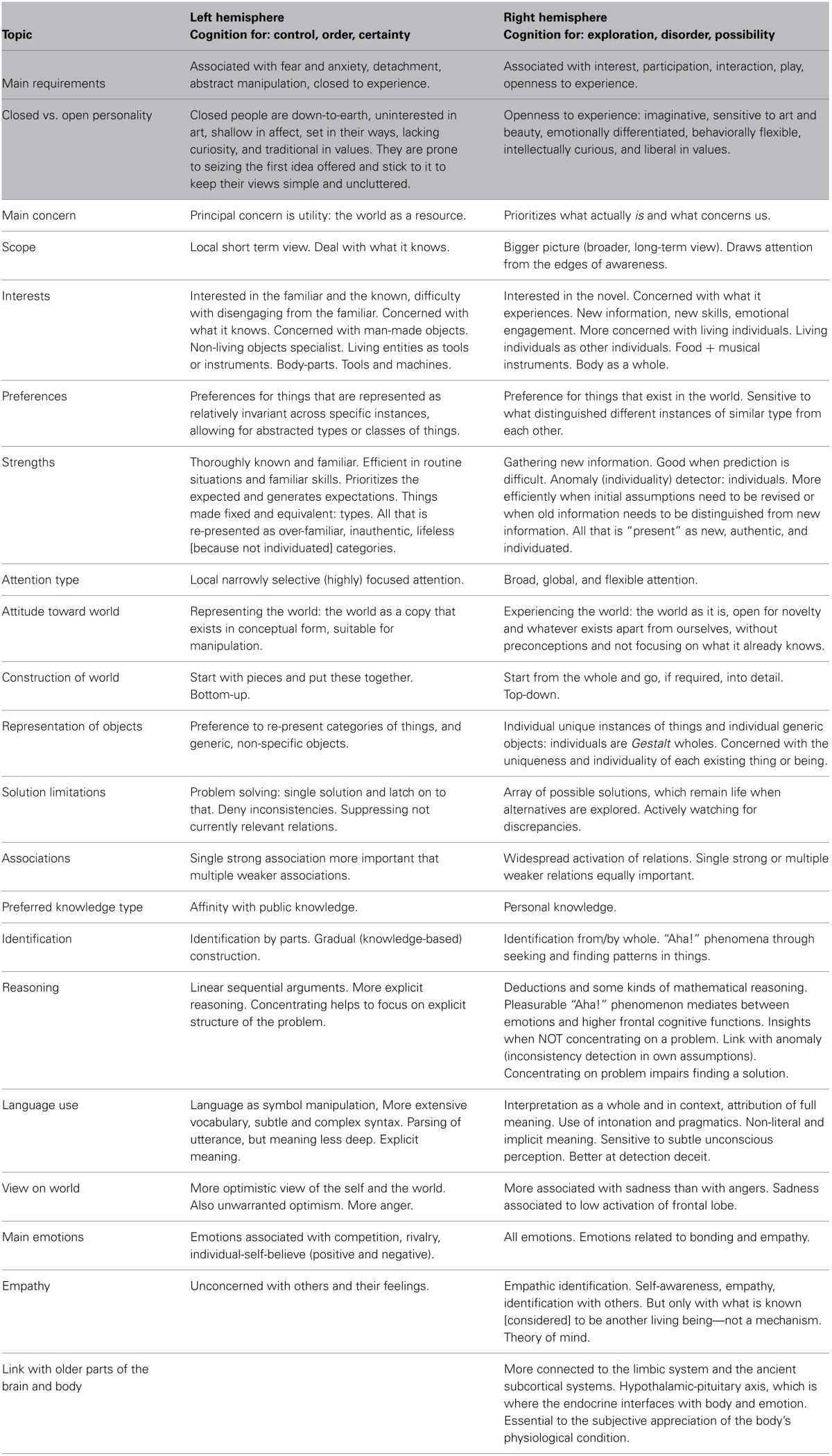
**Overview of roles and approaches ascribed to the left and right cortical hemisphere that together define two quite different stances toward the world**.

The description in the three header rows stems from the requirements of cognition for order and disorder. The header of the table summarizes cognition for order and cognition for disorder. The body of the table contains near literal formulations from chapter 1 of McGilchrist (2010).

McGilchrist argues that in the last two or three millennia, our Western societies have become characterized by an ever growing dominance of the left-hemispheric world view that favors a narrow focus over the broader picture, specialists over generalists, fragmentation over unification, knowledge and intelligence over experience and wisdom, technical objects over living entities, control over growth and flourishing, and dependence over autonomy. In his book, called *The Master and His Emissary* McGilchrist argues that the right hemisphere, with its holistic perspective and more intimate relation with the body is the master that tasks its emissary, the left-hemisphere, with focused assignments. However, in our increasingly culturally defined (i.e., more technically structured and less naturally organized) world, where linguistically transmitted shared knowledge has become more important than individually acquired tacit knowledge, left hemispheric strengths seem to have become more beneficial for most of us than right hemispheric strengths.

### In- and external authority

However, and this is essential for our discourse, the left and right hemisphere require quite different conditions to function optimally. The right hemisphere assumes autonomous participation in an open, dynamic, and infinite world of nested dynamical systems that form dynamically stable and continually evolving entities. In this mode of being, truth is defined as accordance with reality and is to be tested by acting out in the world; right-hemispheric knowledge and experiences are essentially subjective. As such this mode of being is particularly effective in situations where new aspects of the dynamics of the world are to be investigated to expand the thought-action repertoire (Fredrickson and Branigan, 2005) and where novel and creative solutions are appropriate.

In contrast, the left hemisphere assumes a closed, static, and finite world in which entities are symbolic, discrete and abstract and in which one is an “objective” observer instead of a participant. In this mode of being, truth is defined as the result of consistent reasoning and consensually agreed on linguistically shared and presented facts. This mode of being is particularly effective in situations in which problems have to be solved or addressed in a detached, rational, standardized, and communicable way. Scientific communication is a typical example of this. Because of this more narrow focus, left hemispheric strategies essentially depend on processes that create and maintain the required closed, static, and finite world: the normative order introduced earlier. We argue that *authorities*—defined as processes or agents that create, maintain, and influence the conditions in which agents exist—fulfill this role. Adequate left hemispheric strategies, we propose, are only possible if either an internal authority, i.e., the right hemisphere, or external authorities ensure that conditions are maintained in which left hemispheric strategies are effective.

In particular we propose that the authoritarian mode of being corresponds to a left hemispheric dominance in combination with a need for external authorities to create and maintain the conditions in which a dominant left hemisphere can function adequately. Libertarianism corresponds to a right hemispheric dominance that is able to provide the proper conditions for left hemispheric functioning. This entails that the authoritarian agent, as the name suggests, is essentially dependent on *external* authorities, while the libertarian agent, again as the name suggests, is free from external authorities because the agent is able to self-maintain the conditions in which both modes of cognition contribute adequately. To put it bluntly, we argue that authoritarianism in adults is a sign of arrested development that limits individual autonomy growth to environments maintained by external authorities.

### Autonomy in two or three steps

In terms of Figure 1, this can be described as an initial right hemisphere dominated inner-loop in which one learns to master the body through playful interaction with the world. The second loop is left hemisphere controlled because one learns from external authorities and through abstracted linguistically conveyed knowledge about the structures of the world. However the purpose of this phase is to learn how to make the mind a useful instrument. If this process succeeds, it allows one to effectively produce intended results in both culturally defined and natural worlds. As such it is a basis for confidence, further exploration, and gradually increasing autonomy through the ability to co-create ever more extended (both in place and in time) environments in which one can self-maintain the condition for adequate functioning. This describes the third (pre-conditioned) loop.

However, when an agent is unable to make the mind into a reliable instrument, the individual is frequently confronted with the inability to produce intended results. And because the left hemisphere is dominant in this phase, one responds in the complexity reducing control mode favored by authoritarians. It is interesting that “power” is defined as “*the ability to produce intended results* (Russell, 1938)*.*” Earlier we summarized Sternberg's definition of wisdom (Sternberg, 1998) as “the ability to produce broadly beneficial intended results while taking the full consequences of behavior into account.” This suggests defining raw power as “the ability to produce intended results *without* necessarily taking the full consequences of behavior into account.” Its is therefore not at all surprising that typical centralized authoritarian organizations such as bureaucracies, governments, large corporations, and the military are always associated with “power” and standardization.

Libertarians do not need the control over the environment provided by these centralist structures and they are, because they made their mind into a reliable tool, not obsessed with reaching intended results (they can do that more often than not). In contrast they are more interested in understanding the full consequences of behavior. This requires a participatory approach in which one learns to discover and predict the innate dynamics of the social, cultural, and natural world without necessarily controlling or curtailing its diversity. On the contrary, working *with* the inherent dynamics of the world is a way to stabilize it (or not to disturb it). We refer to this creative process of moving with the dynamics of the social and natural world as “co-creation”: a product of open-ended development.

In the next section we will argue that external drivers of behavior (functioning as external authority) are associated with extrinsic motivation and left hemispheric strengths, while internal drivers of behavior are associated with intrinsic motivation and as such with learning to co-create and open-ended development. We will use the appraisal of the environment as the link between open-ended development, the two attitudes toward the world, and motivation.

## Motivations

To be motivated means to be moved to do something (Ryan and Deci, 2000). However it is not yet clear *how* states of the world or states of the individual motivate agents to spend their (mind) time in particular ways. We will therefore start this section with some recent results from soundscape research that helped us to formalize the influence of the environment on motivation.

### Appraisal, motivation, and core affect

A soundscape is a perceived sonic environment and soundscape research addresses the role of sounds and sonic environments on individuals and society. In a recent paper (Andringa and Lanser, 2013), addressing how quiet sounds promote and annoying sounds impede health, we analyzed the words people use to appraise sonic environments (Axelsson et al., 2010). Appraisals are “*cognitive evaluations of events that are considered to be the proximal psychological determinants of emotional experience, with different combinations of appraisals corresponding to different emotions*” (Kuppens et al., 2012). Appraisals typically refer directly or indirectly to motivation. Kuppens et al. lists: motivational relevance (“*Is it important?*”); motivational congruence (“*Is it advantageous or disadvantageous?*”); agency (“*Is it caused by others or myself?*”); problem and emotion focused coping potential (“*Can I cope with the situation and with my emotions?*”); future expectancy (“*Is the expected outcome desired or not?*”). Appraising the environment therefore combines motivation, coping capacity, and expectations of the future. As such the appraisal process involves the evaluation of possible (inter)actions with the environment.

**Figure 3:**
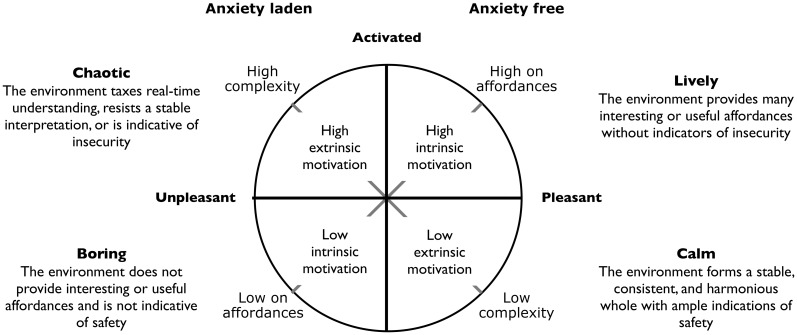
**Core affect, appraisal, and four affective states estimated from soundscape research (Andringa, 2013)**. The main axes reflect the dimensions of core affect, the descriptions in the corners reflect typical appraisals, the description in the circle quadrants reflect four affective states and the diagonal axes represent an alternative way to span the circle in terms of complexity of behavioral selection and affordance content.

Appraisals are also connected to a central concept in emotion theory called “core affect” (Russell, 2003). Core affect is defined as an integral blend of the dimensions displeasure-pleasure (valence) and passive-active (arousal). Unlike emotional episodes, which are relatively infrequent, core affect is continually present to self-report. Core affect is usually visualized as a circle with the pleasure axis horizontally and the arousal axis vertically as depicted in Figure 3. Here relaxed and invigorated moods are situated in the lower and upper right quadrants and moods like boredom and anxiousness in the lower and upper left quadrants respectively. Associated with these moods are calm and lively appraisals on the right and a chaotic and boring appraisals on the left (Andringa, 2013). Appraisals and core affect mutually influence each other (Kuppens et al., 2012).

### Affordances and complexity

Since appraisals involve the evaluation of possible interactions with the environment, they pertain to two main questions: what action opportunities does the environment afford and: how to decide on the best course of action? We will refer to the first question as the affordance content, in Figure 3 as the diagonal from lower left to upper right, and to the second as the complexity of the environment. We will address these issues in order. Because the description of the visual scene leads to quite similar patterns of descriptive words (Russell et al., 1981; Axelsson et al., 2010) we treat our results as if they pertain to perception in general.

Affordances are perceivable action possibilities, provided by an environment (Chemero, 2003) that might be used to satisfy (immediate or future) needs. Affordances arise thus from the interaction of the environment with the perception capabilities of the individual agent. Interesting environments provide discoverable affordances to extend knowledge and skills through, typically, playful interaction (Fredrickson, 1998). Boring environments are devoid of discoverable affordances and do not provide appreciated novelty (e.g., because they are devoid of stimuli, or the stimuli are either too static to be useful or too complex to interpret). The more one interacts (plays) with interesting environments the more complex affordances one learns to perceive.

The complexity of an environment is, in this context, a reference to how difficult it is to cope with environmental challenges and opportunities. Complexity therefore refers not to the environment *per se*, but to the question of how difficult it is *for an agent* to decide on situationally appropriate behavior. Low complexity environments are highly redundant (each part “predicts” the whole, leading to an impression of harmony), which entails that most perceptual evaluations of the environment lead to a similar overall interpretation of pervasive safety. In “calm” low complexity environments action outcomes are relatively insensitive to the details of action selection and action execution; one is neither forced nor enticed to act overtly and the mind is free to wander and to attend its own business (Andringa and Lanser, 2013).

In contrast, highly complex environments are less redundant; for example because of a lack of internal coherence due to a multitude of uncorrelated processes, giving an impression of chaos and unpredictability. This entails that the focus of attention needs to be chosen and adapted well to ensure a proper selection and execution of coping behavior. In contrast to low complexity environments, complex situations may force one to act in a highly controlled fashion and in response to particular events. This entails that action outcomes in complex environments are highly sensitive to detail.

This analysis suggests four qualitatively different types of (sonic) environments in terms of the complexity of action selection and affordance content. The complexity depends on the agent's ability to select a safe course of action. Highly complex or chaotic environments are difficult to interpret (e.g., due to an overabundance of diverse stimuli), actively indicative of insecurity, or in other ways requiring a precise selection of activities. This type of environment activates highly focused mind-states aimed at coping with the here and now. A boring (sonic) environment is low on useful (audible) affordances and is, for that reason, not indicative of safety, which activates alert mind-states. In contrast, a lively environment is not indicative of insecurity and represents many affordances that provide ample interesting opportunities to attend, and it allows one freedom to address the available affordances at will. The fourth environment is calm or relaxing because it provides ample indications of safety and allows as such full freedom of mind-states to relax and recuperate. Figure 3 provides these four domains of environmental appraisal.

In terms of the spiraling open-ended development depicted in Figure 1, the growing ability to detect and effectively use affordances is a measure of progress along the spiral. Initially the affordance content is predominantly used to determine situationally appropriate conformist behavior, but gradually the affordances can be used in the more individualized and situationally appropriate fashion characteristic of co-creation. Similarly, any growth of the agents coping ability in Figure 2 depends on an increasing ability to perceive more and more complex affordances and to learn more and more generic and reliable coping strategies.

### Motivation and reward signals

According to Baldassarre's (2011) recent paper, motivations are based on mechanisms that “*drive learning of skills and knowledge, and the exploitation and energisation of behaviors.*” But extrinsic motivations do this “*on the basis of the levels and variations of homeostatic needs detected within the visceral body*,” while intrinsic motivations “*facilitate this, on the basis of the levels and the variations of such skills and knowledge directly detected within the brain.*” This suggests that, according to Baldassarre, motivations are exclusively based on information derived from either the body or the brain: appraisal of the environment plays no (explicit) role. In addition, skills and knowledge derived from extrinsic motivations “*have the adaptive function to produce behaviours that allow the regulation of those homeostatic needs so as to increase fitness.*” In contrast “*intrinsic motivations have the adaptive function to allow organisms to learn skills and knowledge without the necessity to have a direct impact on homeostatic needs and fitness at the time of the acquisition. These skills and knowledge contribute to increase fitness as they can later be used to learn, relatively quickly, complex behaviours and long chains of actions that regulate homeostatic needs.*”

Strictly interpreted this entails that extrinsically motivated behavior only occurs after the visceral body develops a homeostatic need, while intrinsically motivated behavior has no direct benefit. Consequently a well-fed agent on the track of an approaching train might be fascinated by the complex behaviors and long chains of actions afforded by this experience, but it will not move unless it timely develops a visceral need such as thirst. Yet apart from the absent role of situational appraisal there is much to agree with in Baldassarre's definition. In particularly the role of the perceived needs of the visceral body—now or in the foreseeable future—that define extrinsic motivations and its reward function.

Ultimately, extrinsic motivations are deficiency motivations and are associated with what Maslow referred to as D-cognition (D = deficiency) which he defined as “*the cognitions that are organized from the point of view of basic needs or deficiency-needs and their gratification and frustration {Maslow:^1962^tn, p. 189*}.” The reward signal of D-cognition is need-gratification: the pleasures of food after abstention, restoring order after chaos, relief after a negotiating a dangerous situation, or a monetary reward after boring work. Intrinsic motivations are uncoupled from direct need gratification and allow what Maslow referred to as B-cognition (B = being), a form of cognition in which the world (or objects as Maslow referred to) as it objectively exists can be discovered. These two forms of cognition again refer to the two modes of being outlined in section Two Attitudes Toward a Complex World. It is therefore to be expected that extrinsic motivations are predominantly left hemispheric phenomena, that are driven by utility, while intrinsic motivations are more right hemispheric phenomena associated with exploration and open-ended-learning.

Baldassarre (2011) details how intrinsic motivations provide the reward signals required to drive reinforcement learning. According to him “*intrinsic motivations are based on mechanisms that measure the success of the acquisition of skills and knowledge directly within the brain. For example, these mechanisms drive organisms to continue to engage in a certain activity if their competence in achieving some interesting outcomes is improving, or if their capacity to predict, abstract, or recognise percepts is not yet good or is improving: the brain detects all these conditions without involving the visceral body.*” The mechanisms that measure the successful acquisition of new knowledge, skills, and insights are essentially associated with open-ended development. The experience of this success has been described by Maslow (1954) as a feature of B-cognition. Maslow describes peak experiences as “*feelings of limitless horizons opening up to the vision, the feeling of being simultaneously more powerful and also more helpless than one ever was before, the feeling of great ecstasy and wonder and awe, the loss of placing in time and space with, finally, the conviction that something extremely important and valuable had happened, so that the subject is to some extent transformed and strengthened even in daily life by such experiences.*” According to Maslow the further the development toward self-actualization the more frequent these peak experiences occur, which suggests that they are experienced rewards signals that drive the later stages of open-ended development in B-cognition.

### Motivation, agency, and mind-states

Motivation researchers such as Ryan and Connell (Ryan and Connell, 1989) couple motivations directly to the perceived locus of causality (PLOC), which reflects the degree the individual or some external authority or influence originates the behavior. It is a measure of autonomy and agency. The more autonomous the behavior, the more it is endorsed by the whole self and is experienced as action for which one is responsible (Deci and Ryan, 1987). This leads to a sequence of progressively more agentic motivations: “external,” “introjected,” “identified,” and “intrinsic” reasons to act. According to Ryan (Ryan and Connell, 1989) “*external reasons were those where behavior is explained by reference to external authority, fear of punishment, or rule compliance.*” Introjected reasons are framed in terms of “*internal, esteem-based pressures to act, such as avoidance of guilt and shame or concerns about self and other-approval.*” These are typically situation-enforced motivations with the aim to prevent a worse outcome associated with doing nothing. “*Identifications were captured by reasons involving acting from one's own values or goals, and typically took the form of ‘I want’.*” Through this identification the locus of causality shifts more and more to the agent. Intrinsic reasons for action occur whenever “*the behavior is done ‘simply’ for its inherent enjoyment or for fun*.”

More recently (Malhotra et al., 2008) ordered motivations in terms of intrinsic and extrinsic motivations that have an external or internal perceived locus of causality, and exogenous and endogenous motivation that reflect whether the behavior is driven either by external stimuli or by internal needs or drives. This resulted in four combinations of in-/extrinsic and exo-/endogenous motivations that dovetails very well with the four quadrants in Figure 3 (combining appraisal and core affect), the two modes of cognition in section Two Modes of Cognition, and the role of the two hemispheres described in section Two Hemispheres. As such this allows us to combine many concepts addressed in this paper in a single framework, which is depicted in Table 2.

**Table 2:** **Four motivational states**.

**Motivations**	**Extrinsic**	**Intrinsic**
	Russell: unpleasant	Russell: pleasant
	Ryan: external PLOC, low autonomy	Ryan: internal PLOC, higher autonomy
	Maslow: D-cognition	Maslow: B-cognition
	McGilchrist: left-hemisphere	McGilchrist: right-hemisphere
	Baldasare: extrinsic, deficiency driven, direct fitness benefit	Baldasare: intrinsic, future fitness benefit
	Andringa: no safety, reactive	Andringa: safety, pro-active
**Exogenous**	**Control**	**Exploration**
Russell: highly activated	World: challenging	World: interesting
Malhotra: Driven by external stimuli	Ryan: introjected motivation (internal or esteem-based pressures to avoid harm)	Ryan: intrinsic motivation, completely self-determined activity
	Malhotra: usefulness/utility	Malhotra: hedonistic (fun, enjoyment)
	Andringa: retaining or regaining control	Andringa: learning and playing in safety
	Andringa: high complexity	Andringa: high affordances
	Mind-state: directed attention	Mind-state: flow
**Endogenous**	**Submission**	**Consolidation**
Russell: minimally activated	World: dominating	World: safe
Malhotra: Driven by internal needs/drives	Ryan: external (authority enforced, fear of punishment, rule compliance)	Ryan: identified (personal importances) or integrated (personal goals)
	Malhotra: guided (to external regulation)	Malhotra: self-development, self-enhancement, self-growth
	Andringa: no sense of safety or control	Andringa: restoring resources and caring
	Andringa: low affordances	Andringa: low complexity
	Mind-state: boredom	Mind-state: fascination

This table combines results and concepts from many different domains and provides a generalization of the quadrants in Figure 3.

The entries reflect descriptive words originating from different authors. The upper row and leftmost column reflect descriptions that pertain to the whole row or column respectively. The two rightmost columns, titled extrinsic and intrinsic, reflect modes of being that are directly associated with the two ways to approach complexity, the role of the left and right hemisphere, Maslow's D- and B-cognition, the role of safety in environmental appraisal, and the diverse descriptions of ex- and intrinsic motivations. The two lower rows reflect whether behavior is exogenous and highly activated or endogenous and less activated. The four remaining cells reflect descriptions that pertain to each of the different combination of in-/extrinsic and exo-/endogenous motivation. They also refer to a more general interpretation of the quadrants as depicted in Figure 3. These cells/quadrants have a descriptive name in bold.

The control quadrant reflects a combination of external motivating stimuli with the external perceived locus of causality characteristic of a challenging world. This quadrant reflects a motivational state in which an agent primarily aims to avoid immediate or future injury, harm, or disadvantage. Another name for this quadrant would be the problem-solving quadrant. An agent in this highly complex situation (in terms of behavior selection) is interested in any utility instrumental to avoid negative consequences and to retain or regain control. The associated mind-state is stably focused on the problem as long as the problem exists and is a form of prolonged effortful directed attention (Kaplan, 1995).

The exploration quadrant combines external stimuli with an internal PLOC leading to self-chosen overt behavior that is perceived as fun and enjoyed for its own sake; all characteristic of an interesting world. Aimless but definitely unforced exploration and creation is only possible in apparent safety and requires environmental affordances at a level of complexity that the agent can handle without being taxed too much or too little. The associated mind-state is flexibly focusing on the most interesting aspects of the world, while remaining completely absorbed without lapses and pauses. Flow (Nakamura and Csikszentmihalyi, 2002) is a fitting description for this pleasurable mind-state.

The consolidation quadrant combines individual-need-driven activities with an internal PLOC. This is also only possible in a safe world. This may or may not lead to overt behavior, but is in all situations aimed at unforced self-development, growth, or other forms of psychological and physical recuperation and development. In this quadrant the associated mental activities are free to digress or to wander aimlessly without purpose or goal. One associated mind-state is fascination (Kaplan, 1995) which allows a prolonged, uninterrupted, and effortless immersion in an environment that is pleasant and self-selected to address personal needs proactively. This does not involve directed attention and therefore restores the capacity for directed attention. It is in this mind-state that the mind/brain can address its own needs.

The last quadrant is described with the term submission (to external forces), characteristic of a dominating world. This quadrant is characterized by an external locus of perceived causality in combination with unfulfilled internal needs that offer no other options than to accept guidance, to be subjected to external control (through threat, punishment, or fear), or to do nothing due to cognitive inadequacy given the current environment. In this quadrant the mind is never at rest, but fruitlessly in search of ways to cope. One associated mind-state is boredom, which is described (Martin et al., 2006) as “*Not being in control of life; agitated, yet at the same time, lethargic.*” In addition boredom is associated with restlessness, stress, the feeling of being trapped, frustration, fatigue, lack of concentration, guilt, meaninglessness, and even depression.

The range of scientific domains that have contributed to Table 2 is wide and includes emotion research (Russell, 2003), motivation research (Ryan and Connell, 1989), human machine interfacing (Malhotra et al., 2008), computational development and learning (Baldassarre, 2011), soundscape research (Andringa and Lanser, 2013), personal development (Maslow, 1962), cognitive psychology (Kaplan, 1995), and general cognitive science and culture studies (McGilchrist, 2010). This is an impressive range that is suggestive of the fundamental nature of the topic of this call on open-ended development driven by intrinsic motivations.

## Open-ended development driven by intrinsic motivation

This concluding section returns to the core topic of the call: open-ended development driven by intrinsic motivation. We will use the four motivational states as described in Table 2 to couple motivation to open-ended development via what we call the “open-ended development loop.” We will first address motivation in terms of attitudes and strategies to deal with the world as it is experienced. Secondly we will directly address the intimate relation between open-ended development, intrinsic motivation, and acting out in the world. Thirdly we will outline some consequences for artificial cognitive system research and in particular how to facilitate development toward truly autonomous and moral agents. Finally, we will argue that the left hemispheric biases characteristic of Western cultures have limited artificial cognitive systems research and we suggest a solution to address these limitations.

### Motivation, authority, and co-creation

This subsection returns to the concepts “authority” and “co-creation” that we introduced as essential for open-ended development. We aim to demonstrate that they are important not only as core concepts of cognitive science, but also as defining concepts for agency and even as main forces that shape our (geo)political world.

The section End-State: Self-Actualization and Wisdom, discussed the target of open-ended development and concluded that the authoritarian personality type “seeks, appreciates, and even demands external authorities to maintain the living conditions (the normative order) in which they can function adequately.” In Section In- and External Authority we proposed that the need for external authority was a necessary consequence of left hemispheric dominance that requires a closed, static, and finite world to be effective. This entails that left hemispheric strategies and external authority are mutually dependent: external authorities are expected to maintain the conditions in which the left hemisphere functions adequately. Left hemispheric strategies—through for example intolerance of diversity—reinforce the impact of external authorities through actively allowing external authorities more control while reducing one's own agency. Overall this mode of being reduces the complexity of the world through increased uniformity and shared or centralized authority: the defining characteristics of authoritarians (Stenner, 2005). In moderation this process accounts for the existence of corporations, governments, organized religion, and the military. In excess it leads to stultifying bureaucracies in each of these organizations and eventually to oppressive dictatorship.

However, control through increased uniformity and centralized authority is neither the only nor the best way to deal with a complex world. Section End-State: Self-Actualization and Wisdom concluded that self-actualized or wise individuals feel a moral obligation to contribute to an improved world and we summarized wisdom as “the ability to produce broadly beneficial intended results while taking the full consequences of behavior into account.” Section Authoritarians and Libertarians concluded that the libertarian personality developed the autonomy and skills to co-create living conditions in which (s)he and others feel and act adequately, without the need for external authority to maintain and create these. The driving dynamics for this ability is rooted in the self-confidence resulting from the interest-based exploration and playful behavior that prepared the agent well for an unknown future (Silvia, 2008). In effect this leads to the ability to co-create ever more extended (both in place and in time) environments in which one can self-maintain the condition for adequate functioning, which leads to increasing diversity and individual authority: the defining characteristics of libertarians (Stenner, 2005). This advanced ability characterizes the outer (pre-conditioned) loop of the spiral development in Figure 1.

The difference between the authoritarian and libertarian mode of dealing with a complex world can be (Horney, 1945) summarized as “moving against” or “moving with.” The (according to Horney pathological) “moving against” mode controls diversity and reduces complexity through actively suppressing the inherent dynamics of the world. Note that this is the defining characteristic of our psychology or robotics labs. The (non pathological) “moving with” works *with* or co-opts the inherent dynamics of the world to stabilize it or to prevent the disruption of reliable and useful inherent dynamics. As such “moving with” is a summary of right hemispheric strategies.

The “moving with” mode of being, characteristic of the wise and the self-actualized, allows them not only to create and maintain an individual environment in which they can function adequately, it allows them to co-create the wider environment by gradually reducing the need for external authority (also in others) by (re)allowing and shaping the inherent dynamics of the world in favor of all its inhabitants.

**Figure 4:**
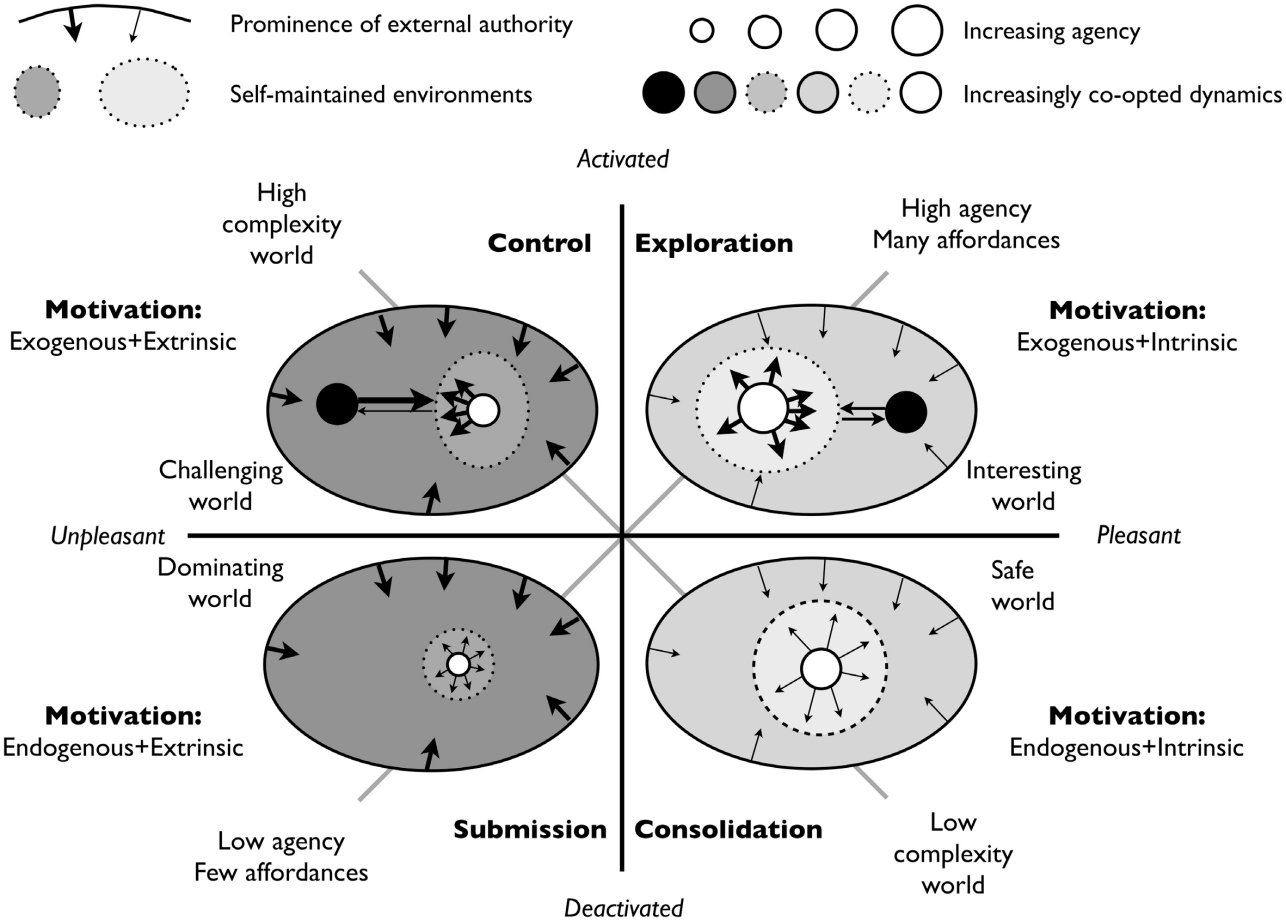
**Motivation, authority, and co-creation**. This figure combines four qualitatively different types of environments in terms of the complexity of action selection and affordance content. The ovals around the agent (white circle) defines self-maintained environments in which the agent can more or less satisfy its needs (light vs. darker shade) and be agentic (size of circles). The dark circle represents a source or novelty approached in danger (left) or safety (right).

Figure 4 provides a graphical depiction of much of the information in Table 2, but it focuses on the relation between the agent and the environment and the difference between external (controlling) authority and internal (co-creating) authority. The large ovals reflect the agent's world that is more (light gray) or less (dark gray) congruent with agentic needs. The prominence of external authorities (the inward pointing arrows) determines whether the world is characterized by suppressed dynamics (the authoritarian mode on the left) or co-opted dynamics (the libertarian mode on the right). The more the agent is able to create and extend a stable agent-maintained environment (dashed oval), the safer and more authoritative it is.

Figure 4 provides the four motivational quadrants defined in terms of the quadrants shown in Figure 3 and Table 2. In the left quadrants the agent is either trying to control or is actually controlled by complex and ill-understood external forces that function as authorities. In the upper left quadrant the agent is challenged by environmental and/or agentic influences which stretch its coping capacity, force it into a narrow range of coping behaviors, and depletes its resources. In the lower left quadrant the agent is part of a world that is mainly beyond its control and understanding, since it does neither afford the agent useful affordances nor resupply of resources. As such it has to accept a minimally agentic role, for example by being forced to participate in activities that may harm its future interests.

In the quadrants on the right the agent's world is congruent with its needs (the most prominent of these is safety). The agent in the upper right quadrant is maximally agentic since it is able to use and explore the affordances of its world in safety and with satisfied basic needs. The agent exists in an interesting world in which it is free to participate in co-creation strategies that gradually elucidates and stabilizes more and more of the world's inherent dynamics for shared benefit. The agent in the lower right quadrant exists in a safe, low complexity environment. It is unforced since, in essence, it profits from earlier co-creation activities of itself and others. This state allows the agent to resupply its resources (to address its needs) and to consolidate its experiences into generalized knowledge and skills.

This then, we conjecture, defines the success of open-ended development: successful open-ended development is characterized by a balance between the co-creation of a low complexity world, in which behavior selection is easy, in combination with high agency due to an abundance of affordances for maintained and extended co-creation. It is this dynamic balance that living agents find highly pleasurable. The enjoyment of successful agentic life—happiness—is therefore deeply meaningful: it is body and mind agreeing on success. And it also suggests that strengths of the right hemisphere, as listed in Table 1, might be understood as pervasive optimization.

### Open-ended development driven by intrinsic motivation

In this subsection we will more directly address the intimate relation between open-ended development, intrinsic motivation, and acting out in the world. In their review paper on extrinsic and intrinsic motivations and their importance for education and development, Ryan and Deci (2000) conclude that “*social contextual conditions that support one's feelings of competence, autonomy, and relatedness are the basis for one maintaining intrinsic motivation.*” They define relatedness as the basic need to feel connected, competence as the basic need to be effective, and autonomy as the basic need to feel agentic. According to Ryan and Deci we need these three basic human needs to be fulfilled in the classroom “*as one is exposed to new ideas and exercises new skills.*”

**Figure 5:**
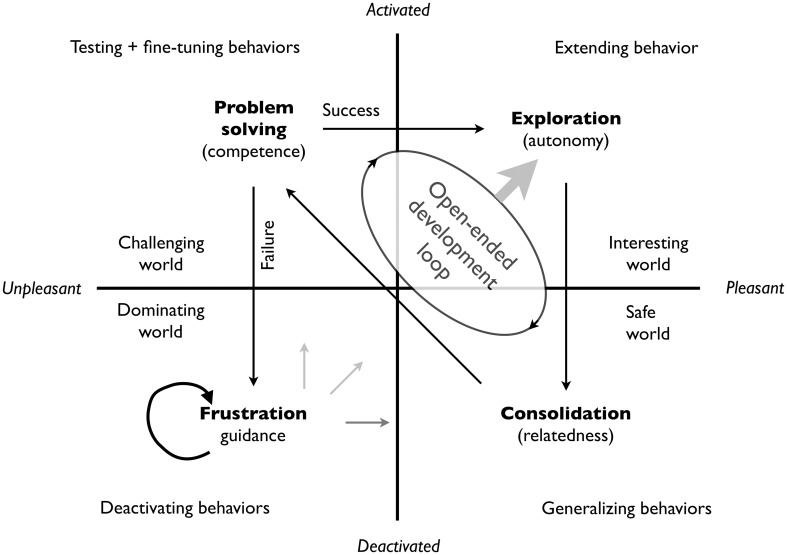
**Open-ended development loop**. The words in brackets originate from Ryan and Deci (2000). The loop depends essentially on the rewards signals associated with exploration (experiencing novelty), consolidation (discovering and fostering relations), and successful problem solving. The reward signals associated with this loop, described as peak experiences (Maslow, 1962), drive the outward spiraling development of Figure 1.

Interestingly, this conclusion can be connected one-to-one with the quadrant structure of Figure 2, Table 2, and Figure 4. In the exploration quadrant one expresses autonomy and agency and extends one's behavioral repertoire. In the consolidation quadrant one develops—in the absence of environmental pressures—new connections between oneself and the environment and one relates and combines hitherto unrelated knowledge and experiences. In doing so one generalizes, stabilizes, and consolidates knowledge and relations (whether mental, social, or otherwise). The consolidated knowledge, (social) relations, and skills, no longer new and unpredictable, become more and more suitable for general utility and in particular problem solving (a left-hemispheric activity). This corresponds to the problem-solving quadrant in which the agent can prove its increased competence and test and fine-tune its extended behavioral repertoire. Successful real-world problem solving leads to confidence, which is a basis for further exploration, consolidation, and testing. This “open-ended development loop” is depicted in Figure 5.

The continuation of the open-ended development loop depends crucially on the success-rate of the in the real-world problem solving ability. Failure to come up with a suitable solution leads to reduced confidence and eventually frustration. Perkins and Hill (1985) provide strong support that boredom is associated with frustration, and since the lower left quadrant is associated with boredom, low agency, and the need for guidance, it makes sense to situate persistent failure and the ensuing low confidence and reduced urge to explore in this quadrant. Persistent failure not only disrupts the open-ended development loop, it is also a strong demotivation to engage in any agentic activity and especially activities that are not habitual (because habits are activated by the environment) and therefore rely on some measure of agency.

This description is reminiscent of the phenomenon of learned helplessness that was discovered when “*dogs exposed to inescapable and unavoidable electric shocks in one situation later failed to learn to escape shock in a different situation where escape was possible* (Maier and Seligman, 1976).” Learned helplessness depends on the uncontrollability of the aversive stimulus, which may entail that the agent learns that its activities do no longer produce intended outcomes. If so the agent does not unlearn its behavior, it simply no longer activates it because of its expected futility. Interestingly, in rats learned helplessness occurs only when one crucial condition is satisfied: “*the response used in the test for learned helplessness must be difficult, and not something the rat does very readily.*” Which, indeed, suggests that learned helplessness occurs only with activities that are agentic. This is the reason why the lower left describes its effect as “deactivating behaviors.”

### Relevance to cognitive system research

We believe that for autonomy to arise in any meaningful way, goal selection and achievement must occur in a (broad) transition region between order and disorder in which both danger and opportunity and defined (conform Figure 2). Without access to such a transition region and the experiences that it affords, the flexible and opportunistic balance and the complementarity between cognition for order en cognition for disorder cannot develop, which entails that there is nothing to drive the open-ended development loop in Figure 5.

Figure 5 suggests a principled way to formulate and structure reward signals because each of the quadrants may be associated with particular reward signals: the lower left with the gratitude of being led or adoration of authority, the upper left with the joy of restoring order, solving problems, or to receive social esteem rewards, the lower right with the joy of insights and understanding and the joys of interpersonal relations (love, friendships, and altruism), and finally the upper left the joy of play, exploration, and creation. The varying states of the environment and the associated appraisals (interesting, safe, or challenging) then bring one in different learning modes. A suitable artificial agent that can engage in this open-ended development loop should be able to learn its way from guided exploration, consolidation, and problem solving into gradually more autonomous exploration, consolidation, and problem solving. In theory, each agent can learn to become autonomous and even wise (i.e., effectively co-creative), as long as it exists in an open environment that offers opportunities for all reward signals.

By constraining the learning environment it is possible to define the character of this agent by ensuring that it is not sufficiently exposed to all reward signals of the open-end development loop. For example, by making it very difficult to continue open-ended learning beyond a certain level of bounded autonomy, one creates an agent who will predominantly experience the reward signals associated with the pleasure of being “moved by.” This will lead to an agent that seeks and loves its servitude.

Alternatively an agent that is “raised” in insecurity will be exposed predominantly to the reward signals and learning outcomes associated with “moving against” uncertainty (e.g., of suppressing diversity), successful problem solving, protocol following, and other forms of cognition for order. This agent will be a quite autonomous apparatchik, someone “*not of grand plans, but of a hundred carefully executed details* (Billington, 1980),” who has no inkling of its role in the grander scheme of things, and who will spontaneously and quite ruthlessly seek, accept, and support external authorities to maintain or restore the conditions for its adequate functioning. Characteristically It will enforce global uniformity and suppress local optimization whenever it increases diversity.

The agent that has been raised in a safe and protected situation and has primarily been exposed to the reward signals associated with love, friendship, and understanding, will develop the many facets of relatedness and profound interest in the world conform the induction capacity of cognition for disorder. This empathic agent will “move toward” others, be comfortable with diversity, and quite able to perceive and understand the beauty and ills of the world. However in times of adversity the empathic agent will not be able to organize or restore and maintain order the way the apparatchik can and it will probably be crushed by its imposed order and intolerance to diversity.

Fourthly the explorative agent is always in search of the reward signals associated with discovery, novelty, creation, and individual expression. This might be an artist agent who seeks the most individual expression of the most individual emotion, a risk-taker, or an autarchic agent that prefers the solitude of self-sufficiency to celebrate its individuality and autonomy. In Horney's (1945) terminology he is “moving away.”

It is interesting that Horney's (1945) terminology—moving away, moving toward, and moving against—fits so well on the three quadrants that define the open-ended development loop. Horney's “moving with” personality, who moves with the dynamics of the world, is her only non-pathological personality. In our framework this is the personality that has learned from all reward signals and that as such has spend much time in the open-ended development loop, with the associated peak experiences. This is the only agent personality who has a proven competence (and autonomy) for most of its existence.

### Including the right hemisphere in cognitive systems research

Gomila's and Müller's (2012) definition of an cognitive system as “*one that learns from individual experience and uses this knowledge in a flexible manner to achieve its goals*” dovetails with how we defined raw power in section Autonomy in Two or Three Steps: “the ability to produce intended results *without* necessarily taking the full consequences of behavior into account.” In that section we concluded that executing raw power is a typical left hemispheric (authoritarian) response. A more developed libertarian, and wiser, response takes the full consequences of behavior into account. This suggests that the left hemispheric dominance of Western societies that McGilchrist (2010) describes has also limited the understanding of the artificial cognitive systems community by focusing its research on left hemispheric strongpoints such as object manipulation, problem solving, and task execution. If so, these Western biases have prevented the artificial cognitive systems community (and other scientific communities) from fully realizing the importance of right hemispheric strengths.

It might therefore be useful to study cultures without these Western limitations. For example Erica Fox Brindley, who studies the intellectual and cultural history of early China (500 BC to 200 AD), wrote a book on individualism in early China [for a summary see Brindley (2011)], which provides a rich description of the roles of agency, autonomy, and authority as the right hemisphere might understand these. She writes for example (Brindley, 2010 pp. xxvii–xxviii):

Earlier Chinese forms of individualism do not generally focus on the radical autonomy of the individual, but rather on the holistic integration of the empowered individual with forces and authorities in his or her surroundings (family, society, and cosmos). For early Chinese thinkers, there is no such thing as unfettered autonomy or freedom of will, in line with Kantian notions of the self. While such concepts are considered problematic even in some Western traditions they nonetheless constitute a core strand of thought that continues to inform contemporary concepts of individualism. In contrast to such conceptualizations, there exists a relative and relational sort of autonomy in early Chinese contexts, a type of autonomy that grants individuals the freedom to make decisions for themselves and to shape the course of their own lives to the fullest degree that they can and should—all from within a complicated and rich system of interrelationships. This type of autonomy, in other words, grants authority to the individual to fulfill his or her potential as an “integrated individual.” The goal of such an individual is to achieve authoritativeness as a person while at the same time conforming to certain types of authority stemming from his or her larger environment.

… Yet the emphasis in the Chines tradition on the relative autonomy of an individual from within a system of holistic and interconnected processes is quite different from many of the models with which we [Westerners] are most familiar. Rather than view autonomy in relationship to a void (individuals as *ex nihilo*), individuals emerge authoritative and powerful as part and parcel of an interconnected web of forces. Therefore, a crucial back-and-forth tug between the self and the various influences and authorities surrounding it is woven in the very fabric of what it means to be a fully attained and empowered individual.

This description, while not even derived from a cognitive science source, illustrates many of the key points of this paper. For example, in terms of agent terminology it states that the goal of a developing agent is to achieve authoritativeness (i.e., to internalize the role of authority) while at the same time conforming to certain types of authority stemming from the larger environment. Since we defined authority as the “processes or agents that create, maintain, and influence the conditions in which agents exist,” this description describes the outward development along the spiral in Figure 1. While all agents influence their living environment, it is the more authoritative—libertarian—agent that successfully can take a role as co-creator and co-maintainer of its environment. So co-creation—defined as working with the inherent dynamics of the world as opposed to frantically controlling and curtailing it—was an inherent part of early Chinese philosophy. In fact it corresponds to the Daoist key term “*Wu wei*,” which “*means something like ‘act naturally,’ ‘effortless action,’ or ‘nonwillful action’* (Littlejohn, 2003).” So the point of open-ended development is to learn “Wu wei” through a process of the internalization of authority insofar achievable given natural laws as highest authority.

The point to make here is not that early Chinese philosophy is an alternative to Western approaches to artificial cognitive systems research, but that our cultural biases limit our understanding. Accounting for these biases and learning from cultures without these particular (and probably other) biases can help to inform the formulation of fundamental research roadmaps such as for artificial cognitive systems. We propose that putting the strengths of the right hemisphere (as summarized in Table 1) center-stage is an essential step to take artificial cognitive systems research out of the closed domain solutions afforded by left hemispheric approaches (and caricatures) of cognitive systems.

If the artificial cognitive systems community indeed tries to rid itself from its limiting biases and adopts approaches that puts the strengths of the right hemisphere and the open-ended development loop central, we have a suggestion for a suitable environment for artificial cognitive system development. This environment offers at the same time (1) many different agents and processes to relate with and care for, (2) many problems to solve and protocols to follow, and (3) an endless and unstoppable variety of novelty and change. This environment might have been an essential progenitor of our cultures because it approximates an ideal balance of reward signals to drive open-ended learning. So a robot that acts responsibly in this environment should be able to acquire the competences and moral development required to function responsibly in the rest of our societies. For that reason we suggest that robot labs should collaborate with … low-tech self-sustaining farms where human, animals, vegetables, fruits, and grains flourish in one of the finest examples of what co-creation can offer.

### Conflict of interest statement

The authors declare that the research was conducted in the absence of any commercial or financial relationships that could be construed as a potential conflict of interest.
